# The development of COVID-19 treatment

**DOI:** 10.3389/fimmu.2023.1125246

**Published:** 2023-01-26

**Authors:** Yongliang Yuan, Baihai Jiao, Lili Qu, Duomeng Yang, Ruijuan Liu

**Affiliations:** ^1^ Department of Pharmacy, The First Affiliated Hospital of Zhengzhou University, Zhengzhou, Henan, China; ^2^ Division of Nephrology, Department of Medicine, School of Medicine, University of Connecticut Health Center, Farmington, CT, United States; ^3^ Department of Immunology, School of Medicine, University of Connecticut Health Center, Farmington, CT, United States

**Keywords:** COVID-19, treatments, antiviral agents, neutralizing antibody therapy, Janus kinase inhibitors

## Abstract

The emergence of severe acute respiratory syndrome coronavirus 2 (SARS-CoV-2) caused a pandemic named coronavirus disease 2019 (COVID-19) that has become the greatest worldwide public health threat of this century. Recent studies have unraveled numerous mysteries of SARS-CoV-2 pathogenesis and thus largely improved the studies of COVID-19 vaccines and therapeutic strategies. However, important questions remain regarding its therapy. In this review, the recent research advances on COVID-19 mechanism are quickly summarized. We mainly discuss current therapy strategies for COVID-19, with an emphasis on antiviral agents, neutralizing antibody therapies, Janus kinase inhibitors, and steroids. When necessary, specific mechanisms and the history of therapy are present, and representative strategies are described in detail. Finally, we discuss key outstanding questions regarding future directions of the development of COVID-19 treatment.

## Introduction

**Figure 1:**
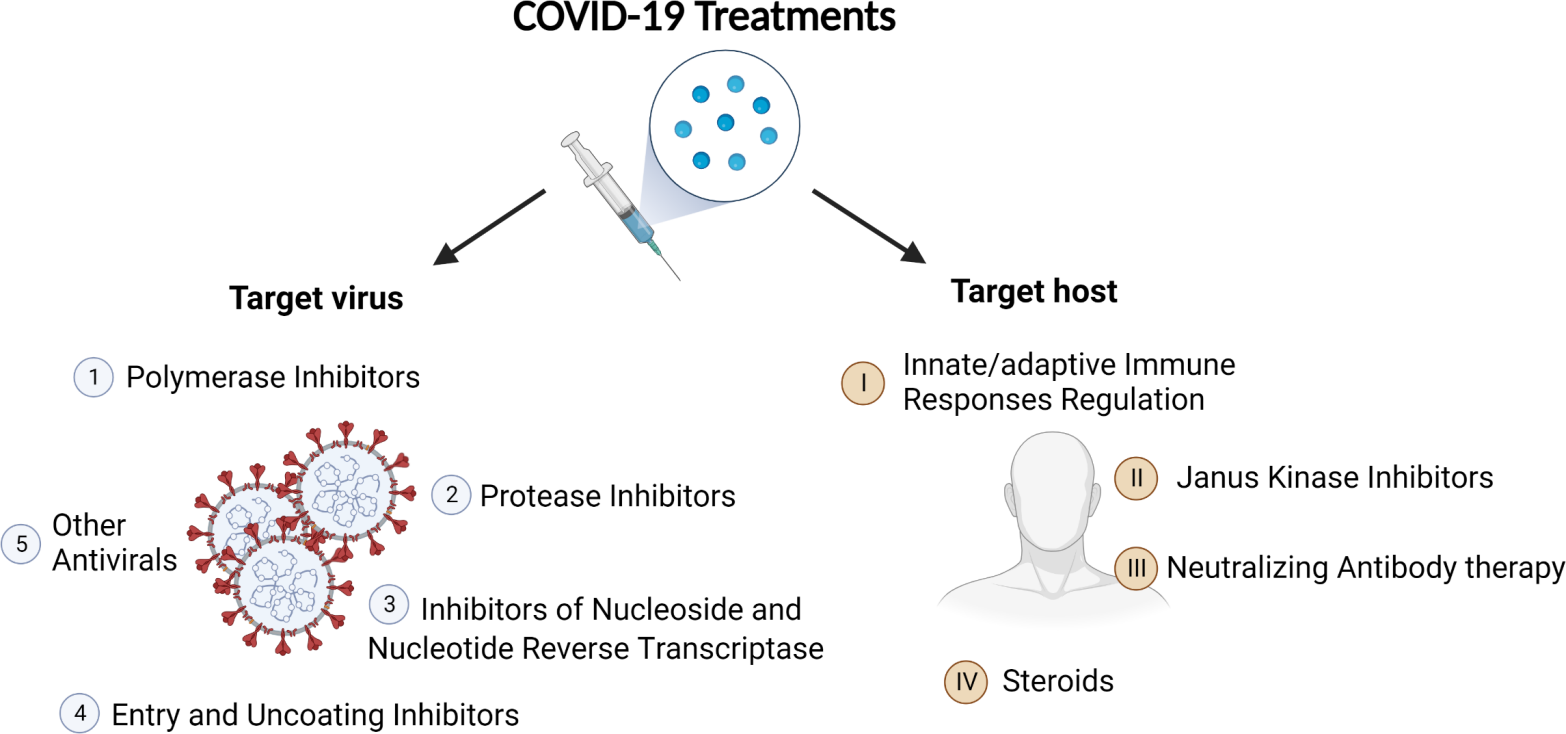
Category of COVID-19 treatments. On the basis of their targets, the treatments can be divided into two big categories: antiviral agents and therapies targeting host.

At the end of 2019, a new coronavirus that quickly causes severe respiratory syndrome and lethal pneumonia emerged in Wuhan, China, and, 3 months later, the World Health Organization characterized the outbreak as a severe acute respiratory syndrome coronavirus 2 (SARS-CoV-2)–induced pandemic that is coronavirus disease 2019 (COVID-19) (1). The pandemic has led to a profound strike on medical care systems, economic progress, and social cohesion around the world. The magnificent research work on developing an effective COVID-19 vaccine has resulted in several safe and effective options (2–4). However, there is still a need to focus on developing potential drug candidates for treating patients with severe clinical symptoms. During the COVID-19 public health emergency, the Food and Drug Administration (FDA) issued Emergency Use Authorization (EUA) for various new drugs and medical products without full FDA approval. Currently, the primary treatments for the disease are antiviral drugs, immunomodulators, neutralizing antibody, and cell and gene therapies (5, 6). Our understanding about the effect of different categories of potential treatments due to their diversity has significantly improved. This study summarized the current therapeutic approaches (**Figure 1**) for COVID-19 with a simple review of SARS-CoV-2 infection pathogenesis, aiming to help researchers and doctors involved in the epidemic to improve their further work.

## Mechanism of COVID-19

Coronaviruses have ignited big-scale pandemics for three times over the past 20 years: SARS from 2002 to 2003, Middle Eastern Respiratory Syndrome in 2012, and COVID-19 emerged at the end of 2019. The COVID-19, caused by SARS-CoV-2, led to an outbreak of unusual viral pneumonia that has spread worldwide and becomes the greatest global public health crisis of this century (7, 8). As of August 2022, there have been over 59 million confirmed COVID-19 cases, and more than 6 million COVID-19–related deaths worldwide. Although a total of 1.2 billion vaccine doses have been administered, COVID-19 is still a huge threat to life due to the lack of effective medical treatment (1).

## SARS-CoV-2 characteristics

CoVs, including SARS-CoV-2, are enveloped viruses with positive-sense single-stranded RNA that possess the largest genomes (~30 kb) among the known RNA viruses, which belong to the Betacoronavirus genus of the family Coroaviridae (9). The genome of SARS-CoV-2 comprises 14 open reading frames encoding nine accessory proteins; four structural proteins of spike (S), envelope (E), membrane (M), and nucleocapsid (N); and 16 nonstructural proteins (nsp1–16). In all these viral proteins, protein S mediates host cell entry of SARS-CoV-2 by direct contact with its cellular receptor–angiotensin-converting enzyme 2 (ACE2). Moreover, the transmembrane protease serine 2 and cathepsin L can also facilitate SARS-CoV-2 cellular entry using a different manner at the plasma membrane surface and endosomal compartments (7, 10, 11). Once enter the host cell, cytosol release of RNA genome of SARS-CoV-2 is activated and starts to replicate. New virions are then assembled and secreted to the intercellular space for infection of neighbor cells (12). SARS-CoV-2 infections often lead to “flu-like” symptoms such as headache, fever, sore throat, backache, cough, and loss of taste or smell. Although plenty of infection cases are asymptomatic or mild, there are certain cases that show severe outcomes and are associated with systemic inflammation, acute respiratory distress syndrome, tissue damage, and cardiac injury. The severe COVID-19 disease with multiorgan damage can be fatal, and its risk largely depends on comorbidities including diabetes, obesity, hypertension, and others (8, 13, 14).

## Host innate immune responses and cytokine storm

Once SARS-CoV-2 invasion happens, the host innate immune response will be rapidly activated followed by the involvement of adaptive immune system (15). As the frontline defense of host, innate immune system employs different strategies for virus detection and elimination to fight against SARS-CoV-2. During the virus infection, the innate immune cells, including natural killer cells, macrophages, monocytes, dendritic cells, and neutrophils, are rapidly recruited and activated first to produce inflammatory cytokines efficiently, such as type I interferons (IFNs) for antiviral activities. Later, B and T lymphocytes are activated for the engagement of immunological memory (16). Host innate immune system primarily relies on pattern recognition receptors (PRRs) to sense virus, bacteria, and other pathogen-associated molecular patterns and/or damage-associated molecular patterns to trigger inflammatory responses that limit viral lifecycle, promote viral clearance, and accelerate the establishment of adaptive immunity (17). The PRRs, expressed by epithelial cells and innate immune cells, are classified into several families: retinoic acid–inducible gene I (RIG-I)–like receptors (RLRs) [including RIG-I and melanoma differentiation-associated protein 5 (MDA5)], Toll-like receptors (TLRs), nucleotide-binding oligomerization domain (NOD)–like receptors (NLRs), the cyclic GMP-AMP synthase (cGAS), absent in melanoma 2–like receptors, and C-type lectin receptors (18). In all the PRRs, intracellular RLR pathways are essential for sensing RNA virus invasion to trigger innate antiviral immune response. Following activation by viral RNA, RIG-I and MDA5 translocate and bind to mitochondria to activate the adaptor protein mitochondrial antiviral signaling (MAVS) that subsequently induces TANK-binding kinase activation and IFN regulatory factor 3 (IRF3) phosphorylation, which, in turn, promotes production of IFNs to prevent virus infection (19, 20). SARS-CoV-2, belonging to single-stranded RNA viruses, can also be detected by MDA5 and RIG-I. However, their roles in regulating SARS-CoV-2 infection seem different (21–24). By screening RNA sensors for SARS-CoV-2 infection in a model of human lung epithelial cells (Calu-3), Yin and colleagues identified MDA5 and LGP2 as the dominant sensors to trigger IFN production in response to SARS-CoV-2 infection (21). Another two independent research groups found that knocking out genes encoding MDA5 or MAVS in human lung epithelial cells leads to impairment of SARS-CoV-2–induced type I IFN production (22, 23). These studies demonstrate the indispensable role of MDA5 in mediating type I IFN expression in response to SARS-CoV-2 infection. However, RIG-I seems to function in a MAVS-IFN–independent way. RIG-I deletion either by small interfering RNA or CRISPR-Cas9 in Calu-3 failed to reduce IFN-β production but still enhanced viral replication (21–23). RIG-I likely restrains full-length ACE2 expression or binds the 3′ untranslated region of the viral RNA genome *via* its helicase domains and consequently restricts cellular entry or replication of virus independently of IFNs (22, 25).TLRs and NLRs also are reported to play roles in anti–SARS-CoV-2 responses. As a classic innate immune signaling, TLRs express widely from tissue cells to innate immune cells, which generally trigger MyD88 or TRIF to transduce signals *via* nuclear factor–κB, mitogen-activated protein kinases, and IRFs to mediate transcriptional activation of pro-inflammatory cytokines (26). Upon SARS-CoV-2 infection, TLR2 senses E protein to enhance inflammatory responses and TLR4 can be activated by S protein to contribute to the release of cytokines (27, 28). In patients with COVID-19, NLRP3 inflammasome activation and its dependent caspase-1 and GSDMD cleavage as well as subsequently IL-1β secretion have been demonstrated, suggesting that NLRs and inflammasome sensors are involved in SARS-CoV-2 infection as well (29–31). In addition, the cGAS–STING signaling pathway, triggered by cytosolic DNA, is also involved in the campaign of fighting against SARS-CoV-2 invasion (32–34). In the case of severe COVID-19, PRRs and cGAS–STING signaling engaged by SARS-CoV-2 induce the expression of both IFNs and numerous pro-inflammatory cytokines, including TNF, IL-6, IL-1β, IL-12, and IL-17 (35, 36). These cytokines act as a two-edged sword, not only aiding in clearing virus infections but also contributing to life-threatening condition caused by cytokine storm. A recent study shows the combination of TNF-α and IFN-γ promotes inflammatory programmed cell death-PANoptosis by activating the Janus kinase (JAK)/signal transducer and activator of transcription (STAT1)/IRF1 axis and caspase-8/FADD signaling (36). The lethal shock phenotype observed in mice administrated with TNF-α and IFN-γ combination mirrors the cytokine storm syndrome in patients with severe COVID-19, emphasizing the link between the dysregulated release of cytokines and the multiorgan damage in patients with SARS-CoV-2 infection (37–39). In addition, the identification of elevated IL-6 levels in serum as a strong predictor of respiratory failure in patients with COVID-19 makes IL-6 one of the critical cytokines in the COVID-19–related hyperinflammatory syndrome (40, 41).

## Adaptive immunity and vaccines

Adaptative immune response, established by activation of T and B cells, sent us a powerful weapon to fight against SARS-CoV-2 pandemic: the vaccine. The activation of innate immune cells during SARS-CoV-2 infection drives T and B cells to respond efficiently to secrete specific antibodies and to kill infected cells, which accelerates the development of acquired immunological memory. The adaptive immunity produced by B and T cells in response to SARS-CoV-2 infection and the vaccines have been discussed in other reviews (42, 43).

## Treatments

All the treatments can be subcategorized into two big groups on the basis of their targets: antiviral agents and therapies targeting host.

## Antiviral agents

Antiviral agents against COVID-19 reported mainly include polymerase inhibitors, protease inhibitors, inhibitors of nucleoside and nucleotide reverse transcriptase, entry and uncoating inhibitors, and other antivirals.

## Polymerase inhibitors

Remdesivir is a nucleotide prodrug, and its active metabolite can inhibit the activity of RNA polymerases, which is a key enzyme for the replication of many viruses, including coronaviridae. Remdesivir showed antiviral effect on SARS-CoV-2 (44, 45), and it was approved by FDA for treating COVID-19. However, the clinical antiviral effect of remdesivir against SARS-CoV-2 remains controversial. One study reported a clinical trial of non-hospitalized patients with COVID-19. Among the enrolled patients, the safety was acceptable following 3 days of treatment with remdesivir, and the risk of hospitalization or death was reduced by 87% compared with the placebo (46). Another clinical trial demonstrated that remdesivir outperforms placebo. The recovery time of adults hospitalized with COVID-19 and lower respiratory tract infection is shortened after receiving remdesivir treatment (47). Whereas, other studies including a multicenter trial conducted in 10 hospitals in Hubei, China, showed that there was no statistically significant difference in the clinical status of patients with COVID-19 receiving remdesivir compared with standard care (48–52). In addition, the researchers also evaluated the effect of baricitinib combined with remdesivir in hospitalized adults with COVID-19. In terms of shortening the recovery time of patients with COVID-19 and speeding up the improvement of their clinical symptoms, remdesivir combined with baricitinib was more effective than remdesivir alone (53).

Favipiravir, an antiviral drug, selectively inhibits the RNA polymerase of viral and has antiviral effects on a variety of RNA viruses (54, 55). A clinical study demonstrated that standard supportive care plus early oral favipiravir monotherapy significantly decreased the recovery time of patients with mild-to-moderate COVID-19 compared with the standard supportive care alone (56).

## Protease inhibitors

Protease is one of the key enzymes in the processing of coronavirus polyproteins. Many studies have been carried out on protease inhibitors for treating COVID-19 in recent years. Lopinavir is a viral protease inhibitor and is primarily used to treat human immunodeficiency virus (HIV). Ritonavir can increase the serum concentration of lopinavir *in vivo* by inhibiting CYP3A-mediated metabolism of lopinavir (57). Therefore, lopinavir/ritonavir is marketed as a combination product. Cao et al. conducted a clinical trial involving 199 hospitalized adult patients with COVID-19. The results showed that lopinavir-ritonavir treatment had no effect on adult patients with severe COVID-19 (58). Moreover, another clinical trial with more participants enrolled at 176 hospitals in the UK was reported subsequently. In this study, 1,616 patients were assigned to lopinavir/ritonavir group and 3,424 patients to the usual care group. Similarly, no efficacy was observed in hospitalized patients with COVID-19 treated with lopinavir/ritonavir (59).

Lopinavir/ritonavir might be more effective if combined with other antiviral regimens. In one study, four patients with COVID-19 were given antiviral treatment, including lopinavir/ritonavir, arbidol, and Shufeng Jiedu Capsule (a traditional Chinese medicine). The pneumonia-related symptoms of three patients were significantly improved; however, the efficacy of the combinational treatment still needs further studies to confirm (60). Moreover, the results of a phase 2 trial showed that early triple antiviral therapy (combined IFN-β1b, lopinavir/ritonavir, and ribavirin) was safe and had a better effect in patients with mild-to-moderate COVID-19 for alleviating symptoms, shortening the time of viral shedding and hospital stay than lopinavir/ritonavir alone (61). Nirmatrelvir is an inhibitor of the SARS-CoV-2 main protease (Mpro) enzyme (62). A phase 2–3 clinical trial was performed in symptomatic, unvaccinated, nonhospitalized adults at high risk for progression to severe COVID-19. In this study, 1,120 patients received nirmatrelvir plus ritonavir therapy and 1,126 patients received placebo. Symptomatic patients with COVID-19 treated with nirmatrelvir plus ritonavir had an 89% lower risk of developing severe COVID-19 than placebo (63).

## Inhibitors of nucleoside and nucleotide reverse transcriptase

Azvudine (FNC), a nucleoside reverse transcriptase inhibitor, has broad-spectrum antivirus activity including HIV-1. FNC had been approved by the national medical products administration (NMPA, China) for AIDS treatment on 21 July 2021. One clinical trial of FNC confirmed that oral FNC (5 mg, qd) could cure patients with COVID-19, and the viral RNA turned negative in about 3.29 ± 2.22 days. The results demonstrated that FNC could be used against SARS-CoV-2 (64). Another clinical trial was performed in China to investigate the anti–COVID-19 effect of FNC. The results of this study showed that FNC could shorten the time of nucleic acid turning negative compared with the standard antiviral drugs for patients with mild and common COVID-19. This work also suggests that FNC is effective for COVID-19, and larger–sample size clinical trials of FNC for treating COVID-19 are needed (65, 66). Recently, NMPA conditionally approved FNC to treat common COVID-19 in adults on 25 July 2022.

Molnupiravir is a small-molecule ribonucleoside prodrug of N-hydroxycytidine and has activity against coronaviruses including SARS-CoV-2 (67). Molnupiravir reduced the risk of hospital admission or death by approximately 50% in nonhospitalized adults with mild-to-moderate COVID-19 who were at risk for poor outcomes (68). To evaluate the efficacy and safety of treatment with molnupiravir in nonhospitalized, unvaccinated adults with mild-to-moderate COVID-19, a phase 3 clinical trial was conducted. Study results suggested that the risk of hospitalization or death of unvaccinated adults with COVID-19 could be reduced by early treatment with molnupiravir (69). Another study showed that molnupiravir was active against the three predominant circulating variants (delta, gamma, and mu) of SARS-CoV-2 and showed a modest antiviral effect (70, 71). Moreover, the UK’s Medicines regulator and the US FDA have authorized the emergency use of molnupiravir for treating mild-to-moderate COVID-19 in adults.

## Entry and uncoating inhibitors

Amantadine can block the early stage of viral replication, which has been used to treat influenza A (72). Amantadine, because of its lipophilic and alkaline physicochemical properties, could cross the lysosome membrane and prevents the release of viral RNA into the cells (73). One study has shown that adamantane may have protective effects against COVID-19. This study has limitations such as a small sample size, and further research is needed to confirm it (74). Enfuvirtide, an HIV-1 fusion inhibitor peptide, could be used as a potent SARS-CoV-2 fusion inhibitor (75). Clinical trials need to be performed to confirm the effect of enfuvirtide against COVID-19.

## Other antivirals

Azithromycin is a synthetic macrolide antibiotic with a broad range of antibacterial, anti-inflammatory, and antiviral properties (76). A prospective, randomized superiority trial done at 19 hospitals in the UK reported that adding azithromycin to standard care treatment did not reduce the risk of subsequent hospital admission or death in patients with mild-to-moderate COVID-19 (77). Moreover, another study also showed that the routine use of azithromycin did not reduce the recovery time or risk of hospitalization for people who were suspected with COVID-19 (78).

Hydroxychloroquine and chloroquine, used to treat malaria and rheumatologic conditions, have been suggested as potential treatments for COVID-19. Currently, at least 80 trials of chloroquine, hydroxychloroquine, or both, sometimes in combination with other drugs, are registered worldwide (79). In one study of 1,561 patients with COVID-19 treated with hydroxychloroquine and 3,155 in usual care, hydroxychloroquine did not lower patient mortality compared with usual care (80). Moreover, hydroxychloroquine did not provide significant improvement in symptom severity for early, mild COVID-19 outpatients (81), and could not prevent symptomatic infection after SARS-CoV-2 exposure (82, 83). In addition, studies had shown that comparing standard care, hydroxychloroquine, alone or with azithromycin did not improve clinical outcomes for patients with COVID-19 (84, 85). Therefore, there is no need to combine azithromycin with hydroxychloroquine in patients with COVID-19.

IFNs, as one of many inflammatory mediators induced by SARS-CoV-2 infection, have been noticed since the beginning of the pandemic, but the effect of IFNs of type I (IFN-I) or type III (IFN-III) families remains controversial (86). Research reported that, unlike other infectious or noninfectious lung pathologies, the expression of INFs was increased in the lower respiratory tract of patients with severe COVID-19. The results indicated that IFNs played opposing roles at distinct anatomical sites of patients with SARS-CoV-2 (86). The hyperinflammation makes the patients succumb rapidly to COVID-19 without the help of IFN. Therefore, IFN improves the prognosis of patients with COVID-19 (87). Kalil et al. found that IFN-β1a plus remdesivir was not superior to remdesivir alone in hospitalized patients with COVID-19 pneumonia (88). Another study showed that IFN-α decreased the mean days of virus clearance and the average days of hospitalization. This study suggests that early administration of IFN-α could be a promising treatment for COVID-19 (89).

## Targeting host

The treatments targeting host include neutralizing antibody therapy, Janus kinase inhibitors, and steroids.

## Neutralizing antibody therapy

The history of antibody therapy can date back to the early 1890s, and, at that time, Dr. Behring and Dr. Kitasato found that the serum from an animal recovered from diphtheria infection could protect diphtheria- and tetanus-infected patients and developed the first serum therapy. Forty years later, serum therapy was widely applied in the treatment of various infectious diseases. However, it had been abandoned with the development of the first antibiotics by the late 1940s. In 1959, a big discovery about the molecular formula of antibodies was made by Gerald Edelman and Rodney Porter (90, 91). Thirty years later, the first antibody “muromonab” was approved in clinics around the world (92). However, the application of the therapeutic antibody was still limited because of the restricted resource at that time. In the 1990s, with the development of antibody engineering technologies (93), the restriction of the antibody resource was broken, which made it possible to check the effectiveness of antibody treatment on a large scale. Since then, antibody treatment has been quickly developed. So far, over 80 therapeutic monoclonal or polyclonal antibodies have been endorsed in the world, which have been mainly adopted in passive immunotherapy. However, with the outburst of COVID-19 and following severe and emergent world health situations, the interest in plasma therapy has been renewed.

## Convalescent plasma

Convalescent plasma from patients who recovered from infection was adopted to treat severe patients. In 2019, the first peer-reviewed study about the effect of convalescent plasma was carried out in China (94). Compared with patients who received standard treatment, 103 patients with severe COVID-19 did not show a statistical difference after transfusion of convalescent plasma. Unfortunately, the trial was halted because of slow and limited enrollment. However, similar studies were carried out in other countries on a smaller scale in 2020. A phase 1 clinical trial study was carried out to inspect the potential of convalescent plasma in Switzerland. Thirty inpatients with COVID-19 were transfused 3 units of 200 ml plasma in 3 consecutive days, followed by comprehensive longitudinal monitoring for over 70 days. The safety of convalescent plasma therapy was confirmed with the absence of transfusion-related adverse events throughout the whole process as a consequence of smaller plasma volumes being adopted in the treatment. Furthermore, faster virus clearance and fewer comorbidities were confirmed in the following monitoring (95). However, this trial was less persuasive by virtue of the small sample size and shortage of control. In later 2020, Spanish scientists did another a higher-scale trial that is multicenter, double-blind, and randomized placebo-controlled. A total of 376 patients with mild-to-moderate COVID-19 with were recruited to receive 250–300 ml of convalescent plasma with high anti–SARS-CoV-2 IgG titers or 250-ml sterile 0.9% saline solution as the control group. The authors did not see a significant viral load decrease and the prevention of progression of the illness at the end of the trial. In addition, one patient showed a serious adverse event after infusion for 7 days (96). At the same time, another trial with 1,181 patients across 23 sites in the US was carried out with the opposite conclusion. This study found that 37 COVID-19–related hospitalization occurred in 589 patients who received control plasma, whereas only 17 of the 592 patients who were infused with convalescent plasma showed disease progression, leading to hospitalization, which means convalescent plasma greatly reduced the hospitalization risk. However, 149 participants among these 1,181 patients were fully vaccinated, and participants older than 65 years only account for 6.8%, all of which made the effectiveness of the treatment controversial (97). Another large convalescent plasma conducted in the UK involving 11,558 patients confirmed that high-titer convalescent plasma treatment failed to improve the survival of hospitalized patients with COVID-19 (98). Estcourt et al. discussed that the reason for the discrepancy between different trials was the standard of patients’ enrollment (99). Moreover, they mentioned another two ongoing trials: the COVID-19 trial and the REMAP-CAP trial, which may bring additional clarity to the effectiveness of convalescent plasma.

## Neutralizing monoclonal and polyclonal antibody therapies

Although convalescent plasma showed partial effectiveness in selected patients, its potential is still controversial. In addition, only part of plasma antibodies will be neutralizing, and those non-neutralizing antibodies will bind to non-spike protein viral antigens, which will sabotage antibody reactions to further cause tissue damage. Indeed, in some convalescent plasma trials, allergic responses and lung damage occurred. Furthermore, the antibody titer in convalescent plasma is low and the resource of the blood is constrained. All these disadvantages restricted the application of convalescent plasma therapy in clinics. In contrast, monoclonal/polyclonal antibodies therapy, as another type of passive immunotherapy, can precisely target the neutralizing sites, and they can be massively produced and easily scalable, which conquers all the disadvantages of convalescent plasma. All these advantages attract medical scientists to put more effort into monoclonal or polyclonal antibodies to develop more potent therapies. As above mentioned, the S protein can bind to receptors for ACE2 to enter host cells. For the early stage, many monoclonal antibody trials targeting S protein were conducted since the routes of the virus entering host cells have been uncovered.

The first monoclonal antibody that was found effective for COVID-19 infection was LY-CoV555. The effect of LY-CoV555 in anti–SARS-CoV-2 infection in nonhuman primates was first reported by Jones et al. (100, 101). In this study, LY-CoV555 (Bamlanivimab) showed strong binding to ACE2 and neutralizing activity. It could reduce viral load in respiratory tract samples even at a low dose. Later, the effectiveness of LY-CoV555 was tested on outpatients and hospitalized patients (102, 103). For the outpatients’ trials, 309 patients were injected with 700, 2,800, or 7,000 mg of LY-CoV555. The viral load of the patients was checked on day 11. Compared with placebo patients, viral RNA showed a significant decrease in the 2,800-mg group patients. In addition, LY-CoV555 decreased the ratio of hospitalization or visiting emergency (102, 104). Two months later, another trial was conducted for the efficacy of LY-CoV555 on hospitalized patients. A total of 163 hospitalized patients were infused with LY-CoV555 and Remdesivir. However, the status of patients was not improved by LY-CoV555 (103, 105). Monoclonal antibodies are restricted only to the same or single epitope due to their monovalent affinity, which may be ineffective against the virus variance. To solve this problem, researchers combined two monoclonal antibodies. Etesevimab, as another monoclonal antibody, was always used jointly with bamlanivimab. A randomized phase 2/3 trial with 613 participants enrolled was carried out at 49 US centers to compare the efficacy of Bamlanivimab as monotherapy or together with etesevimab (106, 107). Bamlanivimab/Etesevimab showed stronger efficacy than bamlanivimab monotherapy in decreasing viral amount in outpatients with mild-to-moderate symptoms. The same conclusion was drawn by another larger trial that bamlanivimab plus etesevimab could decrease the potential of COVID-19–related hospitalization and death (108, 109). Because of their strong efficacy in patients with mild-to-moderate COVID-19, they were issued together for emergency use on 9 February 2021. However, because of the high frequency of the Omicron variant, FDA already retracted this monoclonal antibody for the post-exposure prevention or treatment under the EUA. Later, the study published by VanBlargan et al. also confirmed the futility of LY-CoV555 to Omicron variance (109, 110). So far, the only neutralizing monoclonal antibody issued by FDA for emergency use is bebtelovimab. Iketani et al. confirmed three sublineages of Omicron showed resistance to 17 neutralizing antibodies except for bebtelovimab (111, 112). Westendorf et al. were able to isolate bebtelovimab through a high-throughput B cell screening pipeline. The authors uncovered the LY-CoV1404 epitope is highly conserved in contact residues, which is why they still show neutralizing activity against omicron variance (113, 114). The efficacy of bebtelovimab was also confirmed by another study conducted by Wang et al. and published 1 month ago (115, 116). In this study, they also identified that the Omicron variance showed more transmissible and more evasive to antibodies.

## Janus kinase inhibitors

Serum concentrations of proinflammatory cytokines and chemokines—including IFN-γ, TNF-α, IP-10, G-CSF, IL-2, IL-6, IL-8, IL-9, IL-10, and IL-17—were increased in patients with severe COVID-19 and strongly correlated with disease outcome (117). The JAK/STAT pathway regulates a number of inflammatory cytokines and growth factors by transferring signals from receptors in the cell membrane to the nucleus, leading to hematopoiesis, lactation, and the immune system and mammary gland development (118, 119). JAKs are tyrosine kinases that bind to the cytoplasmic domains of type I and type II cytokine receptors. When ligands bind to their receptors, the intracellular portion of JAKs will be activated, which recruits and phosphorylates STATs. Activated STATs translocate into the nucleus and bind to the promoters of related genes, inducing the expression of specific genes (120–123). These cytokines are highly important in initiating and orchestrating innate and adaptive immune responses but may also be a source of excessive or uncontrolled inflammation and tissue damage in patients with COVID-19. The importance of JAK/STAT pathway in malignancies and autoimmune diseases has been reported (124–131); therefore, inhibition of the JAK/STAT pathway is a promising approach for the treatment of various diseases.

JAK inhibitors can competitively bind to the adenosine triphosphate-binding site of JAKs and interfere with the phosphorylation of STATs proteins, thereby inhibiting the expression of downstream inflammatory genes and growth factors (132, 133). Currently, JAK inhibitors have achieved efficacy in a wide range of immune-mediated inflammatory diseases, such as rheumatoid arthritis (RA) (134), myelofibrosis, and polycythemia vera (135–137). The severity of COVID-19 is strongly associated with SARS-CoV-2–induced hypercytokinemia and inflammation (138, 139). Up to now, some JAKi (such as Baricitinib, Ruxolitinib, Tofacitinib, and Nerizutinib) have had significant clinical impacts on improving clinical outcomes of hospitalized patients with COVID-19. These kinase inhibitors are used as treatments for COVID-19 because they inhibit virus-induced immune activation and signaling of inflammation (140).

## Baricitinib

Baricitinib is a JAK1/JAK2 inhibitor that blocks cytokine and growth factor receptor stimulation, thereby reducing downstream immune cell function (117). Baricitinib is used for the treatment of RA and has shown success in clinical studies in RA. Baricitinib is reliably absorbed when administered orally and is therefore highly bioavailable and well tolerated in patients with RA (141, 142). COVID-19 infection–induced cytokine signaling pathways—including IL-6, IL-2, IL-10, IFN-γ, and GM-CSF—are elevated in hyperinflammatory conditions but are disrupted by baricitinib (53, 117). Furthermore, baricitinib may also have direct anti–SARS-CoV-2 activity by interfering with viral endocytosis, thus hindering SARS-CoV-2 entry and infection of susceptible cells (143).

Numerous studies have established the potency of baricitinib in hospitalized participants with COVID-19. Improved oxygenation and reduced levels of systemic inflammatory cytokines have been reported in patients with COVID-19 treated with baricitinib (144–146). A meta-analysis of randomized controlled trials shows that treatment with JAK inhibitors (including baricitinib) in hospitalized patients with COVID-19 can significantly reduce the risk for COVID-19 death by 43%, whereas it led to a significant decrease in the risk for mechanical ventilation or ECMO (extracorporeal membrane oxygenation) initiation by 36% (147). A study demonstrated that baricitinib plus standard of care including corticosteroids predominantly reduced mortality at 28 days and 60 days in patients who were receiving National Institute of Allergy and Infectious Disease (NIAID) ordinal scale score 7 population (NIAID-OS 7; hospitalized and on IMV or ECMO). Overall, 28-day all-cause mortality among patients on IMV or ECMO at baseline was 58% among those receiving placebo and 39% among participants receiving baricitinib. Sixty-day mortality was significantly lower in the baricitinib group compared with that in the placebo (45% *vs*. 62%, respectively) (148). In addition, another two studies showed that baricitinib absolutely reduced mortality in patients with moderate-to-severe or severe COVID-19 (149, 150). Another meta-analysis of randomized controlled trials evaluated the safety and efficacy of baricitinib in hospitalized patients with COVID-19 and showed that baricitinib could lead to better clinical outcomes for hospitalized patients with COVID-19 (151).

Since baricitinib’s continued success in COVID-19 clinical studies, FDA issues the first EUA for baricitinib in combination with remdesivir for the treatment of hospitalized adults with COVID-19 and pediatric patients 2 years or older requiring assisted invasive mechanical ventilation or ECMO. The EUA, revised on 28 July 2021, to address safety concerns and protect public health, authorizes baricitinib as a stand-alone therapy. In May 2022, baricitinib was approved by the US FDA for the treating hospitalized adults with COVID-19 requiring supplemental oxygen, mechanical ventilation, or ECMO.

## Tofacitinib

Tofacitinib, an orally available JAK inhibitor, can inhibit inflammatory cytokines such as IL-2, IL-4, IL-6, and IL-7 through inhibiting JAK3 and JAK1, which is approved for treating autoimmune diseases and RA (152–155). Many studies suggest that tofacitinib therapy can be continued in patients with COVID-19.

A double-blind, interventional phase 3 trial (NCT04469114) including 289 patients evaluated the safety and efficacy of tofacitinib in hospitalized patients with COVID-19 with pneumonia (156). The cumulative incidence of death or respiratory failure through day 28 was 18.1% in the tofacitinib group and 29.0% in the placebo group. Specifically, 28-day all-cause mortality was significantly lower in the tofacitinib group than in the placebo group (2.8% vs. 5.5%, respectively) (156). In another study by Roman and colleagues on 62 patients with severe COVID-19, it has shown that tofacitinib group showed lower mortality and the incidence of admission to intensive care than the control group (16.6% *vs*. 40.0% and 15.6% *vs*. 50.0%) (157). Furthermore, an ongoing randomized, double-blinded, placebo-controlled phase 2 study developed by Yale University evaluated the safety and efficacy of tofacitinib in 60 hospitalized patients with COVID-19 (18–99 years old) who require supplemental oxygen and have serologic markers of inflammation but do not need mechanical ventilation (NCT04415151). Analysis of these RCT studies demonstrated the promising efficacy of tofacitinib in mortality and the incidence of invasive mechanical ventilation.

## Ruxolitinib

Ruxolitinib, a JAK1 and JAK2 protein kinase inhibitor, can suppress cytokine production associated with myelofibrosis, polycythemia vera, and acute graft-versus-host disease (158–163), which is similar to SARS-CO-V2 infection. The antiviral potency of ruxolitinib has also been shown to be effective against HIV and Epstein–Barr virus infection (164, 165). Since ruxolitinib has been successfully used to treat the diseases associated with hyperimmune syndrome, it has been employed in patients with COVID-19.

Several studies are being conducted with ruxolitinib in several clinical settings. In a global, double-blind, placebo-controlled, 29-day, multicenter phase 3 trial, 432 patients were randomly assigned to receive ruxolitinib (n = 287) or placebo (n = 145) plus the standard of care to assess the efficacy and safety of ruxolitinib in hospitalized patients with COVID-19. In this study, the primary objective was missing: the composite endpoint was 12% in both ruxolitinib-treated patients and placebo-treated patients; the mortality rate by day 29 was 3% in patients receiving ruxolitinib, compared with 2% in the placebo group. In addition, 8% ruxolitinib-treated patients had received invasive ventilation compared with 7% of 145 placebo-treated patients, and 11% ruxolitinib-treated patients had received ICU care compared with 12% placebo-treated patients (166). Another phase 3 study was conducted to assess the efficacy and safety of ruxolitinib with 5 mg bidaily or 15 mg bidaily in patients with COVID-19–associated ARDS who require mechanical ventilation. There was no statistically significant improvement in mortality in ruxolitinib group at day 29 compared with that in the placebo. However, it is significant when US study participants were analyzed separately, and when data from both treatment arms were pooled, it could also be detected for the overall population (167). In addition, a beneficial effect of ruxolitinib was reported in COVID-19 pneumonia in a multicenter study with 43 participants. In this study, 90% of ruxolitinib-treated patients showed computed tomography improvement at day 14 compared with 61.9% of placebo-treated patients. Three patients in the control group died of respiratory failure, with an overall mortality rate of 14.3% on day 28; no patients in the ruxolitinib group died. This study demonstrates that ruxolitinib tends to improve the clinical status of patients with COVID-19 in a shorter time (168).

## Nezulcitinib

Nezulcitinib, an inhaled, lung-selective, pan-JAK inhibitor, has shown strong, broad inhibition of JAK/STAT signaling in the respiratory tract in experimental mouse models, which is a promising therapy for broad intervention in excessive immune response in the airways. Therefore, nezutinib, administered by nebulization, may provide a new therapeutic modality to inhibit cytokine release and decrease morbidity and mortality in patients with COVID-19 with acute lung injury. A phase 2 clinical trial with inhaled nezulcitinib 3 doses (1, 3, and 10 mg once daily for 7 days) explored the safety and efficacy in 25 patients with severe COVID-19 and initially reported trends toward improving the clinical status and decreasing mortality in patients requiring supplemental oxygen (169). The lower mortality rate and earlier clinical recovery in patients receiving nezulcitinib compared with that in patients receiving a placebo suggest that it is a promising therapy for cytokine-driven lung inflammation.

## Steroids

The latest research shows that SARS-CoV-2 infection can motivate the immune system and ignite inflammation, which occasionally causes lethal cytokine storm. Corticosteroids have been used to treat inflammation related diseases in the last decade, such as RA and asthma. However, the trials of corticosteroids in patients with COVID were not encouraged in the beginning considering their function in suppressing the immune system. After several randomized clinical trials, corticosteroids have been proved to be able to improve survival in severe COVID-19 (170), which will be discussed in the following part.

## Dexamethasone

In 2020, a controlled and open-label trial, including around 6,425 hospitalized patients, that lasted for 28 days was started in the UK (171). A total of 4,321 patients receiving usual care were taken as control, whereas another 2,104 patients received dexamethasone treatment. After 28 days, dexamethasone decreased the death rate of patients rely on invasive mechanical ventilation or oxygen. Another clinical trial stated that intravenous dexamethasone was able to increase ventilator-free days in patients with COVID-19 with moderate or severe ARDS in comparison with standard care alone (172). However, long-term treatment with 12 or 6 mg of dexamethasone for 10 days in patients with COVID-19 with severe hypoxemia did not show improvement in mortality or health-related quality of life (173). Similarly, another clinical trial study that lasted for 10 days showed that dexamethasone at 12 mg/day compared with that at 6 mg/day did not decrease the death rate of patients with COVID-19 and severe hypoxemia in the absence of life support (174), whereas a 90-day follow-up of which revealed the benefits of the high dose compared with that of the low dose (175). Furthermore, an open-label and randomized clinical trial demonstrated that high-dose dexamethasone, compared with low-dose, improved clinical symptoms within 11 days in hospitalized patients with COVID-19 who rely on oxygen therapy (176).

## Budesonide

The study carried out in Spain and Argentina stated that inhaled budesonide was safe and could reduce the incidence of severe syndrome in inpatients with COVID-19 (177). Another similar study was accomplished in the UK community, which showed that inhaled budesonide could promote the recovery rate and reduces hospitalization or death in patients with COVID-19 (178). In addition, a phase 2 randomized controlled trial indicated that administration of inhaled budesonide in early COVID-19 could reduce the chance of worsening, which may be due to its inflammatory modulating effect through improving T-cell response (179, 180).

## Ciclesonide

CONTAIN phase 2 randomized controlled trial exhibited that patients with inhalation and intranasal ciclesonide showed less severe symptoms than the patients in placebo groups, which did not show in young patients. However, further research is required due to insufficient evidence (181). Another randomized clinical trial of the efficacy of inhaled ciclesonide in outpatients showed that ciclesonide did not reduce the recovery time in adolescents and adult patients (182). Nevertheless, it is still early to get a conclusion about the effect of ciclesonide.

## Glucocorticoids

Hydrocortisone and methylprednisolone all showed positive effects in patients with COVID-19 with severe symptoms revealed by two clinical trials (183, 184). Prednisolone showed a protective effect in patients with post–COVID-19 diffuse parenchymal abnormalities, which did not show dose dependency (185).

## Conclusions

**Figure 2:**
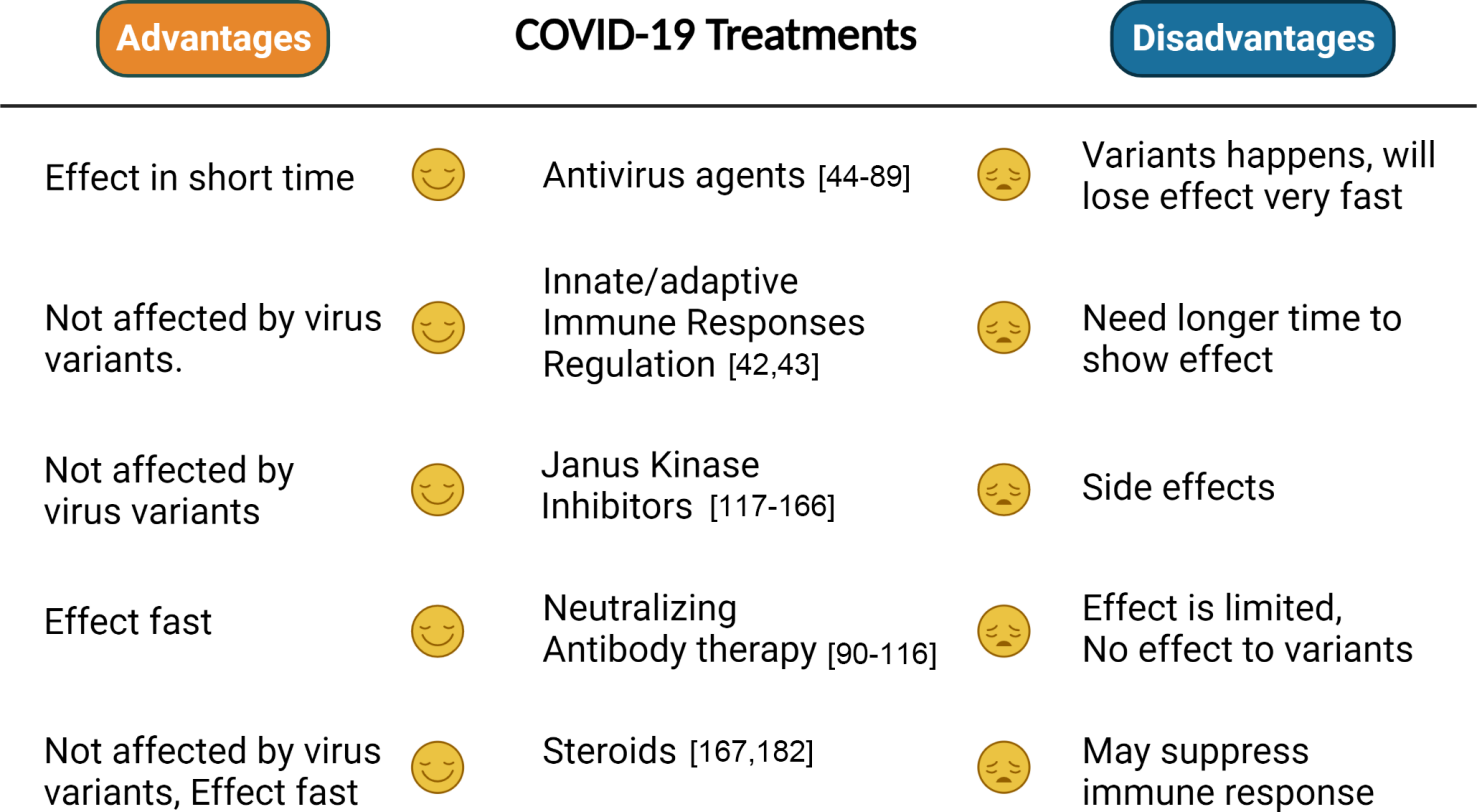
Comparison between different treatments. The advantages and disadvantages of each treatment are summarized on the basis of whether they will be efficient to variants, have side effects, or effect fast.

COVID-19, as a worldwide spreading virus with high transfection and lethal, caused millions of people to die, exposing the weakness of the human healthcare system in coping with emergent and serious health risks. All the above-illustrated treatments with different advantages or disadvantages (**Figure 2**) have been developed, targeting the virus or the host, which indeed saved lots of lives before effective vaccines were produced. Although some trials failed, they still played positive roles in the human healthcare development. The effect of many treatments is compromised or controversial, such as the antiviral agents and neutral antibody therapies, which may be caused by viral variants, and combinational treatment can be considered based on the results of some clinical trials. Many studies reported combinational therapies showing better effects than single treatment, such as molnupiravir/fluvoxamine/Paxlovid, IFN-β1b/lopinavir/ritonavir/ribavirin, and lopinavir/ritonavir/arbidol/Shufeng Jiedu Capsule. In general, combinational therapy showed more advantages than single treatment. In addition, compared with antiviral agents and neutral antibody treatments, JAK inhibitors show more promising effects, which may be worthy to continue in future studies. For the effect of steroids, it is still early to get a conclusion. Although current vaccines restrain the development of the invisible “war” ignited by COVID-19, humans should be getting ready anytime for future pandemic threats that may jeopardize the whole world. It is impossible for humans to predict the next pandemic, which makes it impractical to prepare effective vaccines in advance. However, effective treatments targeting symptoms caused by infection are still significant, with respect to being prepared for another potential pandemic threat. Hence, more efforts are still needed to be put into developing effective treatments for COVID-19.

## Author contributions

YY and DY arranged an overview of the content. All authors contributed to the article and approved the submitted version.
